# Paroxysmal Nocturnal Dyspnea Secondary to Right Ventricular Myxoma: A Novel Presentation of an Unusual Tumor

**DOI:** 10.1155/2018/4791379

**Published:** 2018-02-28

**Authors:** Tristan E. Knight, Bruce Shiramizu, Princeton Ly, Karen S. Thompson, Venu Reddy

**Affiliations:** ^1^Division of Pediatric Hematology and Oncology, Department of Pediatrics, Children's Hospital of Michigan, Detroit, MI, USA; ^2^Division of Pediatric Hematology and Oncology, Department of Pediatrics, John A. Burns School of Medicine, University of Hawaii, Honolulu, HI, USA; ^3^Department of Pediatrics, David Geffen School of Medicine, University of California Los Angeles, Los Angeles, CA, USA; ^4^Department of Pathology, John A. Burns School of Medicine, University of Hawaii, Honolulu, HI, USA; ^5^Clinical Laboratories of Hawaii, Honolulu, HI, USA; ^6^Division of Pediatric Cardiology, Department of Pediatrics, John A. Burns School of Medicine, University of Hawaii, Honolulu, HI, USA

## Abstract

A 14-month-old male presented with paroxysmal nocturnal dyspnea and grade III/VI systolic ejection murmur at the upper left sternal border with an S4 gallop and was subsequently found to have a right ventricular cardiac myxoma. Prior presentations of these tumors have been with exertional syncope and murmur, asymptomatic murmur, or exertional dyspnea; the presentation of such a tumor with paroxysmal nocturnal dyspnea is novel.

## 1. Introduction

Primary pediatric cardiac tumors are rare; ventricular myxomas are rarer still. Moreover, their presentation is varied, making detection and early diagnosis difficult. The purpose of this case report is to review prior presentations within the pediatric population and to present a novel presentation of such a tumor with paroxysmal nocturnal dyspnea.

## 2. Case Report

**Figure 1:**
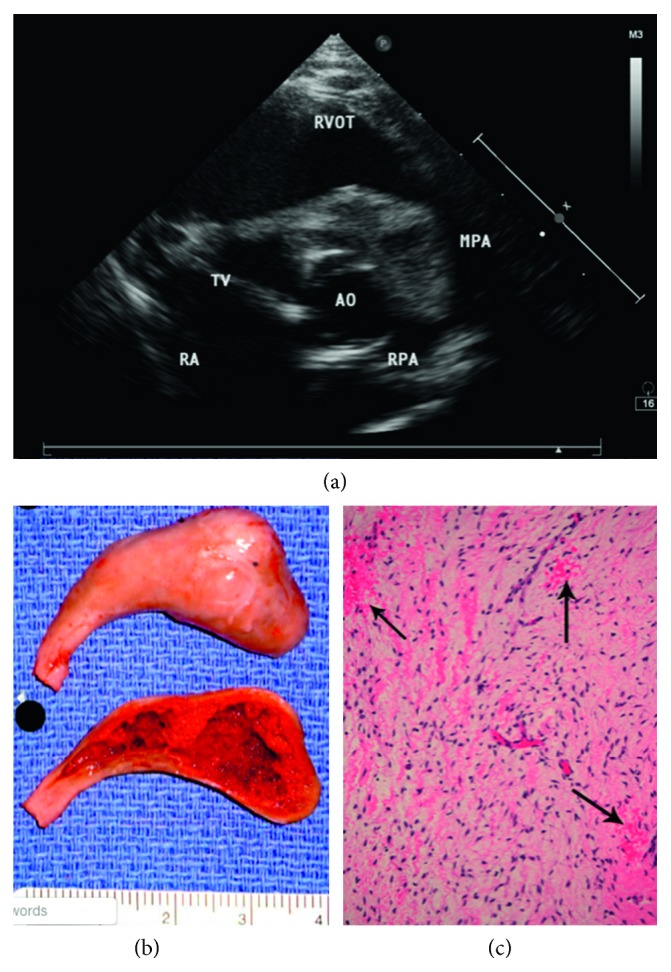
Echocardiogram (a) shows an evident pedunculated mass, attached to the base of the anterior leaflet of the tricuspid valve and right ventricular septum extending into the right ventricular outflow tract. Gross pathology (b) shows a resected tumor, measuring 3.8 × 2.4 × 1.5 cm with infarction and degenerative changes. (c) H&E stain, 200x: stellate fibroblasts displaying small, elongated, dense nuclei and eosinophilic cytoplasm with indistinct cell borders enmeshed within a myxoid stroma. Extravasated red blood cells are seen adjacent to capillaries (arrows).

A 14-month-old male presented with 3 months of worsening paroxysmal nocturnal dyspnea, with multiple sleep interruptions consisting of episodic respiratory distress, cyanosis, and coughing. These episodes worsened in the 2 weeks prior to presentation, occurring up to 3 times nightly. Medical and family/social history was noncontributory, with native-Hawaiian background and normal growth. Vital signs were appropriate, and exam was normal other than a grade III/VI systolic ejection murmur best appreciated at the upper left sternal border with an S4 gallop. Electrocardiogram showed sinus tachycardia, right axis deviation, and right ventricular hypertrophy but was otherwise unremarkable. Sedated, supine 2-dimensional echocardiogram with Doppler showed a pedunculated mass with an estimated size of 4.3 × 2.0 × 1.4 cm occupying most of the right ventricle, attached to the base of the anterior leaflet of the tricuspid valve and right ventricular septum. The mass extended into the right ventricular outflow tract and caused marked obstruction of the pulmonary artery during systole (Figure 1). Right ventricular pressure was significantly elevated between 100 and 110 mmHg based on tricuspid regurgitation jet; positional evaluation was not performed. Left ventricular morphology, size, and function were normal.

The mass was subsequently resected. Intraoperatively, the tumor base was located at the leading edge of the anterior leaflet of the tricuspid valve, with some involvement of the chordae tendineae and the papillary muscles. Maximal resection was attempted without creating a defect in the tricuspid valve, leaving some areas of fibrotic, grossly abnormal tissue in an attempt to recreate physiologic anatomy. Postoperatively, 2-dimensional echocardiogram was repeated, showing absence of the previously present mass, a competent, nonregurgitant tricuspid valve with some thickening of the anterior leaflet, and normalization of right ventricular pressure. Postoperative electrocardiogram was unchanged and did not show any abnormal cardiac rhythms.

**Figure 2:**
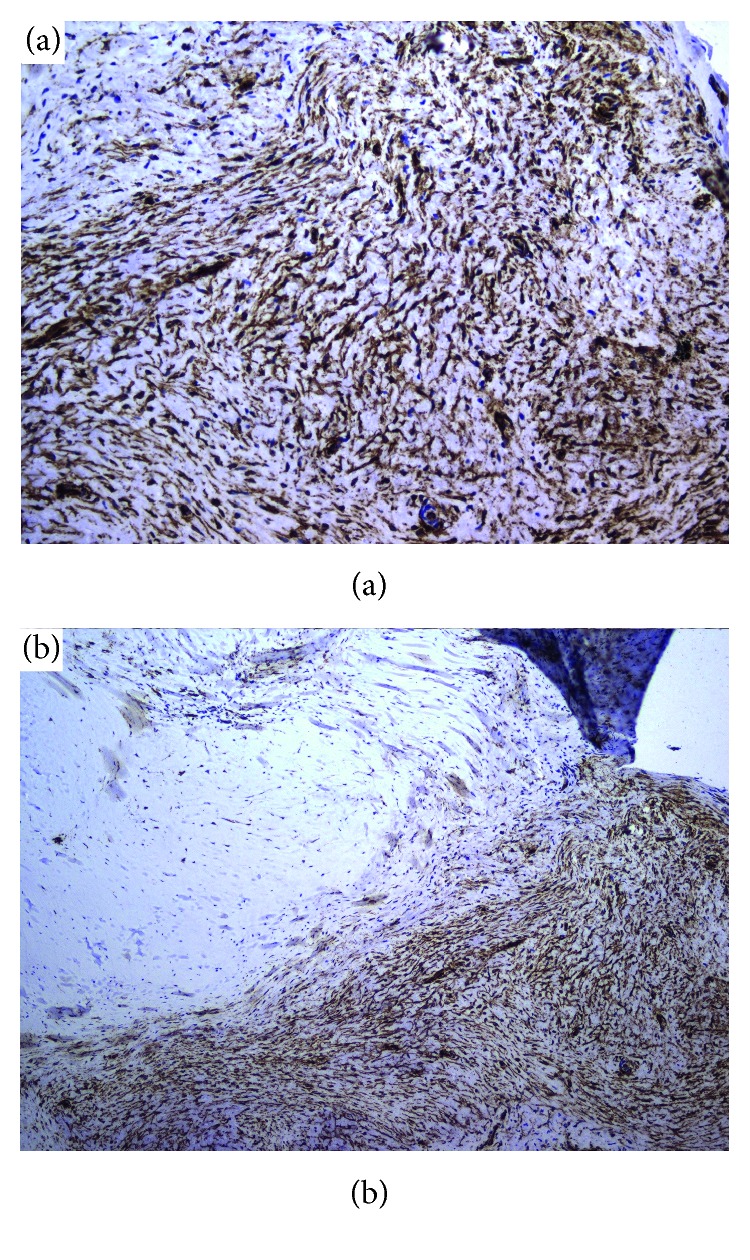
(a) Strongly positive CD34 staining in tumor sections under high magnification. (b) Low-power view shows strong CD34 staining in the tumor cells (lower right) with adjacent negatively staining cardiac tissue (upper left).

Grossly, appearance was of a tan pedunculated mass measuring 3.8 × 2.4 × 1.5 cm, with a tan-gray, smooth, firm surface and infarction/degenerative changes. On cross section, a central cavitary area was noted, with fibrous material adherent to the inner lining and an average wall thickness of 0.8 cm (Figure 1). Microscopic sections revealed fibroblasts within a myxoid matrix and small, dense, elongated nuclei with inconspicuous mitoses and no atypia (Figure 1). Immunohistochemical staining was strongly positive for CD34 (Figure 2(a)) and negative for CD31, desmin, and smooth muscle actin. Sections demonstrated a successful transition between lesion and normal cardiac tissue at surgical margins (Figure 2(b)). The final diagnosis was ventricular cardiac myxoma. Postoperative course was uneventful, and the patient was discharged home well; as of writing, he has experienced complete resolution of prior symptoms and subsequent echocardiograms have been normal, without evidence of recurrence.

## 3. Discussion

Primary pediatric cardiac tumors are rare, with an autopsy incidence of 0.0017–0.28% [1]. 25% are malignant and 75% are benign, with secondary cardiac tumors (mostly metastatic Wilms tumor and leukemic chloromas) being 10–40 times more common than primary [1]. Rhabdomyomas are the most common primary cardiac tumors (50%), followed by fibromas (25%) and myxomas (9–15%) which, despite being the most common adult cardiac tumor, are rare among children [1]. Rhabdomyomas are derived from the myocardium and extend into the cardiac cavities. They may cause arrhythmias secondary to disruption of myocardial conduction and rarely occur individually, typically being associated with tuberous sclerosis [2]. Fibromas are derived from cardiac mesenchyme and occur sporadically; in contrast to rhabdomyomas, they remain confined to the myocardium without extending into the cardiac cavities [2]. Myxomas are derived from primitive connective tissue and occur almost exclusively within the atria. They are quite friable and are also more likely to be pedunculated; they are therefore capable of causing both intermittent intracardiac obstruction as in our patient and embolic phenomena, including pulmonary embolism and embolic stroke [3]. A small but important subset of myxomas occur as part of the Carney complex—a rare, autosomal-dominant multiple neoplasia syndrome, classically characterized by lentiginosis, endocrine tumors, and cardiac myxomas [4]. Immunohistochemical staining (strongly positive for CD34 and negative for CD31, desmin, and smooth muscle actin) supports a diagnosis of cardiac myxoma (typically staining positive for CD34 and negative for smooth muscle actin [5]), while arguing against that of fibromas (typically staining positive for smooth muscle actin and negative for CD34 [6]) or rhabdomyoma (typically desmin and actin-positive [7]).

There is a general paucity of reports on ventricular myxomas in the pediatric literature. However, the presentation of such a tumor with paroxysmal nocturnal dyspnea is novel; prior cases have presented with exertional syncope and murmur [8, 9], asymptomatic murmur [10–12], and exertional dyspnea [13]. Due to their friability, atrial myxomas can present with ischemic stroke, pulmonary embolism, and sudden death [3, 14]. Though not as-yet described among ventricular myxomas, this presentation could theoretically occur. The differential diagnosis associated with paroxysmal nocturnal dyspnea typically includes interstitial lung disease, hypertrophic cardiomyopathy, and left-sided heart failure; ventricular myxoma tumor is not one of these differentials. However, as shown here, a diagnosis of ventricular myxoma should be considered in pediatric patients with paroxysmal nocturnal dyspnea and murmur with an intraventricular mass, and referral to cardiology urgently sought with removal as early as possible.
